# The capacity of young national medicine regulatory authorities to ensure the quality of medicines: case of Rwanda

**DOI:** 10.1186/s40545-022-00492-2

**Published:** 2022-11-24

**Authors:** Jean Baptiste B. Shabani, Egide Kayitare, Eric Nyirimigabo, Vedaste Habyalimana, Marilyn M. Murindahabi, Lazare Ntirenganya, Clarisse Irasabwa, Eugene Rutungwa, Jose Edouard Munyangaju, Innocent Hahirwa

**Affiliations:** 1 EAC Regional Centre of Excellence for Vaccines, Immunization, and Health Supply Chain Management, College of Medicine and Health Sciences, University of Rwanda, Kigali, Rwanda; 2 Rwanda Food and Drugs Authority, Kigali, Rwanda; 3 School of Medicine and Pharmacy, College of Medicine and Health Sciences, University of Rwanda, Kigali, Rwanda; 4 School of Business, College of Business and Economics, University of Rwanda, Kigali, Rwanda

**Keywords:** Poor-quality medicines, Falsified medicines, Substandard medicines, Medicines regulatory practices, Healthcare supply chain, Rwanda FDA

## Abstract

**Background:**

Access to quality medicines is a global initiative to ensure universal health coverage. However, the limited capacity of National Medicines Regulatory Authorities (NMRAs) to prevent and detect the supply of poor-quality medicines led to the predominance of sub-standard and falsified (SF) medicines in the supply chains of many countries. Therefore, this study was designed to assess the capacity of a young NMRA to ensure the quality of medicines with Rwanda as a case study.

**Objective:**

This study aimed to assess the capacity of the Rwanda FDA, a young NMRA, to identify gaps and existing opportunities for improving regulatory capacity and ensuring the quality of medicines.

**Methods:**

This study used a descriptive cross-sectional design with both quantitative and qualitative approaches. The quantitative approach used a self-administered questionnaire to collect data from employees of Rwanda FDA who are involved in medicine regulatory practices based on their positions while the qualitative research approach covered a desk review of key regulatory documents. The data collection tool was developed from the World Health Organization (WHO) Global Benchmarking Tool (GBT) for “Evaluation of National Regulatory System of Medical Products Revision VI”.

**Results:**

Of the 251 WHO sub-indicators assessed, 179 sub-indicators (71%) were fully implemented, 17 sub-indicators (7%) were partially implemented, 9 sub-indicators (4%) were ongoing and 46 sub-indicators (18%) were not implemented by the time of the study. The results of the study showed that the estimated maturity level at which Rwanda FDA operates is maturity level 2. The study reported the challenges hindering the implementation of key regulatory functions that need to be addressed. Challenges reported include but are not limited to understaffing, lack of automation system, poor implementation of the quality management system, lack of screening technologies for SF medicines, low capacity of the quality control laboratory to test all sampled medicines and lack of regulatory inspection tools/equipment.

**Conclusion:**

Findings indicated that all key regulatory functions were operating and supported by the legal framework. However, the implementation of key regulatory functions faced challenges that need to be addressed for better organizational effectiveness and compliance with the requirements of a higher maturity level.

## Background

The quality of medicines should be the priority of every country to ensure that the public has access to quality, safe, effective and affordable medicines [1]. However, this is not the case, as some 2 billion people worldwide cannot access quality medicines [2]. The World Health Organization reports that 10.5% of the medicines on the market are of poor quality, being either sub-standard or falsified (SF) [3].

SF medicines are more prevalent in low- and middle-income countries (LMICs) with an overall prevalence of 13.6% and African countries have the highest prevalence of poor-quality medicines in LMICs, at 18.7% [4]. The cause of the infiltration of poor quality medicines into the supply chain, especially in low- and middle-income countries has been associated with factors such as inadequate funding, lack of skilled personnel, lack and ineffective regulatory systems and frameworks [5].

The presence and use of SF medicines have many implications for both public and the economy of the country. Firstly, SF medicines in the supply chain prevent people from accessing quality, safe and affordable medicines [1]. Secondly, the use of poor-quality medicines by the public leads to the waste of limited resources, antimicrobial resistance, prolonged illness and death of people [6]. Thirdly, the overall consequence is the increase in the costs associated with interventions and initiatives put in place to address problems caused by poor-quality medicines [1, 7].

To ensure the quality of medicines on the market in any country, a stable and well-functioning national medicine regulatory authority (NMRA) must be in place [8]. WHO states that a well-functioning NMRA shall have the capacity to perform all key regulatory functions including Medicines registration and marketing authorization, Vigilance, Market Surveillance and Control, Licensing Establishments, Regulatory Inspections, Laboratory Testing, Clinical Trials Oversight, and Lot Release [8]. However, a study on Medicines Regulation in Africa has shown that most NMRAs in Africa have little capacity to perform all key regulatory functions [9]. In East African Community, SF medicines have been reported in all member states [10].

To assess the capacity of any NMRA in ensuring the quality of medicines, the WHO uses the Global Benchmarking Tool (GBT) to evaluate the National Regulatory System, which classifies the NMRA into maturity levels (ML) from 1 to 4 showing weak to strong regulatory NMRA [8]. A weak NMRA has been reported as the main factor enhancing the infiltration of SF medicines into the market in most African countries [11]. Among EAC countries, Rwanda has a young NMRA, which was established in 2018 [12], and countries without or with young NMRA might face challenges of poor-quality medicines [13].

Rwanda Food and Drugs Authority (Rwanda FDA) was established with the mandate to protect public health from defective and SF medicines through transparent regulation of regulated premises and products [12]. Strengthening such capacity of NMRA requires a time of development and implementation of policies, laws, regulations, guidelines, standard operating procedures, capacity building of human resources, and infrastructure [8].

However, the information and the evidence about the capacity of Rwanda FDA to exercise its mandate and functions are limited. Therefore, this study aimed to assess the *capacity of the young national medicine regulatory authority established in Rwanda to ensure the quality of medicines based on the* World Health Organization (WHO) Global Benchmarking Tool (GBT) for “Evaluation of National Regulatory System of Medical Products Revision VI”.

## Methods

### Study setting

The study was conducted in Rwanda Food and Drugs Authority (Rwanda FDA) premises located in Nyarutarama Plaza, KG 9 Avenue, Gasabo District, Kigali City and its quality control laboratory located in Kicukiro District, Kigali City near the building of Rwanda Standard Board compound at the road KK 15 Rd, 49.

### Study design and period

This study was descriptive and cross-sectional that used a mixed approach combining qualitative and quantitative research components. The quantitative research used a self-administered questionnaire while the qualitative research approach consisted of a desk review of key regulatory documents including policies, laws, regulations, guidelines, procedures, reports and lists of registered products. The data collection tool was developed from the World Health Organization (WHO) Global Benchmarking Tool (GBT) for “Evaluation of National Regulatory System of Medical Products Revision VI”. The study was conducted from November 2021 to January 2022.

### Study population

The targeted study participants were 51 employees of Rwanda FDA who have expertise in medicine regulatory practices based on their roles in ensuring the quality of medicines. The targeted population was composed of staff working in divisions/Units having in their responsibilities to ensure the quality of medicines including Human Medicine and Devices Assessment & Registration Division, Food and Drugs Import & Export Control Division, Food and Drugs Inspection & Compliance Division, Pharmacovigilance & Food Safety Monitoring Division, Quality Control Laboratory Division/Medicines and Cosmetics Testing Unit, and Administration). All 51 participants were involved in the study.

### Data collection tools

The data collection tools included a self-administered questionnaire and a review checklist for a desk review focused on key regulatory documents including policies, laws, regulations, guidelines, procedures, reports and lists of registered products, lists of licensed establishments and the number of GMP inspections conducted.

The self-administered questionnaire comprised the section on demographic data profile and the section on the implementation level of key regulatory functions as independent variable. It was organized according to the research topic to ensure the research questions and objectives are answered and determined, respectively.

### Data collection procedure

The methodology used in the study was based on WHO/GBT to evaluate the capacity of any NMRA that has nine functions, nine indicators and 268 sub-indicators that have to be assessed to determine the maturity level of any NMRA [8]. In this study, eight functions, nine indicators and 251 sub-indicators were assessed. This is because one function having 17 sub-indicators was not assessed. The non-assessed function is the National Regulatory Authority (NRA) lot release, which is a function established specifically for the regulatory release of specific biological products, including vaccines not yet implemented by Rwanda FDA as a young NRA [8].

To assess the capacity of NMRA the following rating scale is used for every sub-indicator [8]: not implemented which means that there is no evidence of the regulatory document/process required. Ongoing implementation means that the NMRA drafted the regulatory document required or put in place the process required but not yet followed in the implementation. Partially implemented means that the NMRA has the required document/process that has been implemented for less than 2 years. Implemented means that the NMRA has the required document/process in place used for at least 2 years and it can show the track record of applying it.

The data were collected using a questionnaire and a review of key regulatory documents. The questionnaires were given in person by the researcher to respondents. The documents reviewed were accessed from Rwanda FDA website and in-house sources including databases and reports. Written informed consent was obtained from each participant before completing the questionnaire.

### Data analysis

The data entry was done using Microsoft Excel. These data were exported into Statistical Package for the Social Sciences (SPSS) version 16.0 for further analysis. Descriptive statistics were used to analyze the socio-demographic data, implementation levels of functions and indicators, responses and results are presented in the tables.

## Results

### Socio‑demographic characteristics of participants

Most of the study respondents were aged between 25 and 34 years (51%) and the majority of the respondents were male (71%). The majority of the respondents were Bachelor’s degree holders (90%). Regarding the work experience of the respondents, the majority was in the range of 1–5 years (59%). All respondents received basic training related to the responsibilities of the position occupied (Table 1).

**Table 1:** Socio‑demographic characteristics of participants

Characteristics	Frequency	Percentage (%)
Age		
Below 25 years	1	2
Between 25–34 years	26	51
Between 35–45 years	24	47
Total	51	100
Gender		
Male	36	71
Female	15	29
Total	51	100
Level of education		
Bachelor’s degree (A0)	46	90
Masters’ degree	4	8
Ph.D	1	2
Total	51	100
Work experience		
Between 1–5 years	30	59
Between 5–10 years	19	37
Above 10 years	2	4
Total	51	100
Basic training related to the responsibility of the position received		
Yes	51	100
No	0	0
Total	51	100

### Implementation of functions assessed

From a total of eight functions assessed in the study, the sub-indicators related to MA and LT functions were the most implemented, with 83% and 82%, respectively, while RS and RI were the least implemented functions, with 62% each. 179 sub-indicators (71%) were fully implemented out of 251 assessed sub-indicators (Table 2).

**Table 2:** Implementation of functions assessed

Function	# Sub-indicators	Implementation of sub-indicators			
		Fully	Partially	Ongoing	Not
Registration and marketing authorization (MA)	35	29 (83%)	4 (11%)	0 (0%)	2 (6%)
Laboratory testing (LT)	28	23 (82%)	0 (0%)	2 (7%)	3 (11%)
Licensing establishments (LI)	19	15 (79%)	0 (0%)	1 (5%)	3 (16%)
Vigilance (VL)	26	20 (77%)	4 (15%)	0 (0%)	2 (8%)
Market Surveillance and Control (MC)	27	20 (74%)	2 (7%)	0 (0%)	5 (19%)
Clinical Trials Oversight (CT)	30	19 (63%)	3 (10%)	5 (17%)	3 (10%)
National Regulatory System (RS)	60	37 (62%)	4 (6%)	0 (0%)	19 (32%)
Regulatory inspection (RI)	26	16 (62%)	0 (0%)	1 (4%)	9 (34%)

### Implementation of indicators assessed

From a total of nine indicators assessed in the study, the sub-indicators related to legal provisions, regulations and guidelines were the most implemented, with 93% while the sub-indicators related to monitoring progress and assessing outcomes and impact were the least implemented, with 29% (Table 3).

**Table 3:** Implementation of indicators assessed

Indicator	# Sub-indicators	Sub-indicators implemented	% of sub-indicators implemented
Legal provisions, regulations and guidelines	59	55	93
Organization and governance	18	15	83
Regulatory process	49	39	80
Resources	42	32	76
Policy and strategic planning	12	9	75
Leadership and crisis management	5	3	60
Transparency, accountability and communication	28	13	46
Quality and risk management system	14	6	43
Monitoring progress and assessing outcomes and impact	24	7	29

### Estimated maturity level at which Rwanda FDA operates

Based on the number of sub-indicators implementation at different maturity levels and considering the maturity level algorithm as defined by World Health Organization [14], the findings of this study showed the estimated maturity level at which Rwanda FDA operates is maturity level 2 (Table 4).

**Table 4:** Estimated maturity level at which Rwanda FDA operates

Maturity level	# Required sub-indicators fully implemented	Study results	# Required sub-indicators PI or OI	Study results	# Required sub-indicators NI	Study results
1	27	26	0	1	0	0
2	53	53	3	3	0	0
3	183	174	14	23	0	0
4	240	179	11	0	0	49

## Discussion

This study aimed to assess the capacity of young national medicine regulatory authorities to ensure the quality of Medicines and Rwanda was considered as a case study.

The results of this study indicated that the legal provisions in place give the Authority the powers to formulate regulations and guidelines; grant, suspend or withdraw authorization; seize or confiscate products not conforming to the provisions of the laws, and establish a tariff for services rendered by Rwanda FDA; impose administrative sanctions arising from breach of those legal provisions [12]. Rwanda FDA has a well-defined structure [15], however, this structure does not include the structure of quality management systems [16, 17].

As Rwanda FDA has no quality management system (QMS) structure in place it is difficult for the authority to implement a comprehensive QMS that integrates risk management principles. This is consistent with the findings of the assessment conducted by WHO in 26 NMRAs of sub-Saharan African countries which found that four of the 26 NMRAs (15%) were found to have some elements of QMS [18]. However, this is contrary to the WHO guidelines on the implementation of QMS for NMRA [17]. A possible explanation for this could be that Rwanda FDA is a recent NMRA since it was established in 2018.

The quality management system (QMS) shall be established and implemented by any NMRA to ensure that each operation/activity is carried out to a defined and uniform standard. The QMS shall ensure that each step of the regulatory process is identified, documented and monitored [19].

The attainment of International Standard (ISO) certification especially ISO-9001 Quality management systems—Requirements by the National Medicines Regulatory Authority is an indicator of a well-functioning regulatory authority [20]. Interestingly, the management has appointed QMS focal persons in each division/unit.

Medicines registration and marketing authorization function were found to be supported by legal provisions. The results of the present study are similar to the results of the WHO report dated 2010 which assessed the regulatory capacity of 26 African countries [18]. A possible explanation for these results may be the leadership’s commitment to being a world-class regulatory authority.

The defined registration timeline to register the medicines was stated to be 9 months in the guidelines but the findings of the present study showed that the timeframe for product registration can go beyond the required timeframe. These findings were also reported by MTaPS which conducted a study in seven African countries and two Asian countries to evaluate the registration systems of medicines used in maternal, newborn and child healthcare [21]. One of the solutions to the lengthy registration process may be the strengthening of the existing harmonization of medicines regulation in the East African Countries [10, 22] and the proposed African Medicines Agency (AMA) [9, 23].

The same findings showed that Rwanda FDA has a working national pharmacovigilance system supported by the law, regulations and guidelines to perform its functions. The findings are in accordance with the findings of the study conducted by Barry et al. [24] in a comparative assessment of PV systems in East Africa that found that the PV system was supported by legal instruments and guidelines. These results may be explained by the fact that the national PV system in Rwanda was overseen by the ministry of health before the creation of Rwanda FDA.

Under-reporting of ADRs/AEFI was among the challenges reported in this study. This result corroborates the findings of Isah et al. [25] and Ampadu et al. [26].

The results of this study showed that a remarkable step forward has been achieved in Post-Marketing Surveillance since the creation of Rwanda FDA. This result corroborates the findings of Ndomondo-Sigonda, et al. [9] who conducted a study on medicines Regulation in Africa to evaluate the current state and opportunities. These results may be explained by the existence of the PROFORMA project, a 5-year project that aims to strengthen the National pharmacovigilance infrastructure and post-marketing surveillance systems in Ethiopia, Kenya, Tanzania, and Rwanda [27].

The results of the study revealed that the method used to prevent and detect SF medicines at the port of entry is not adequate, as only visual inspection is performed to verify label requirements and package integrity, storage conditions, dosage units and documentation according to import or export requirements and SF medicines screening technologies/tools are not used to prevent and detect SF medicines before they enter the market. The same findings were also reported by Roth, Biggs, Bempong, who found that the use of screening technologies is not widely implemented in LMICs to combat SF medicines [28].

The findings of this study showed that public and private manufacturers, distributors, wholesalers, importers/exporters as well as retailers shall possess an operational license issued by Rwanda FDA. It is well documented in the legal provisions that an inspection for confirmation of compliance with good practices is required to grant or re-grant a license or approval of a substantial modification. However; it was found that there was insufficient staff to conduct the regulatory inspections, especially on-site GMP inspection, virtual GMP inspection or GMP desk review assessment. These findings reflect those in a study conducted in Angola in the department of pharmaceutical inspection [29] that found that the inspection department was lacking staff to conduct regulatory inspections. These findings are rather disappointing because they may hinder the registration process and affect the availability of quality-assured, safe and efficacious medicines in the Rwandan market. Regulatory inspection is a key regulatory function in any NMRA because it reveals deficiencies and potential errors in the manufacturing process, quality control, storage and distribution of medicines [8].

The quality control laboratory does not have ISO 17025 Quality Management System (QMS) accreditation for testing and calibration services and is not the WHO Guidance for Good Laboratory Practice Prequalified. These findings broadly support the WHO resolutions on the regional strategy for the regulation of medical products in the African region of 2016 documented that, 34 countries in sub-Saharan Africa (72%) have quality control laboratories in place which are at different stages of development and most of them lack a QMS and adequate equipment for quality control laboratories [30].

The results of this study showed that the quality control laboratory is not operating at the required level of stringency. It is not ISO 17025 certified nor WHO Guidance for Good Laboratory Practice Prequalified which are the global reference tools for measuring the performance standards of quality control laboratories to ensure the quality of medicines throughout the supply chain [31].

## Limitations, strengths and future research

This study used the data collection tool developed from the World Health Organization (WHO) Global Benchmarking Tool (GBT) for “Evaluation of National Regulatory System of Medical Products”, thus the data presented here can be comparable if similar studies are conducted in other countries that have young *national medicine regulatory authorities*. However, it provides an overview of Rwanda FDA capacity and does not delve into each regulatory function. Therefore, future research conducting a deep analysis of each functionality is encouraged.

## Conclusions and recommendations

The findings of this study showed that there is a progressive improvement in the regulatory capacity of the Rwanda FDA to ensure the quality of medicines in Rwanda since its inception. The legal framework is in place to enable effective and efficient implementation of the key regulatory functions that include Registration and Marketing Authorization, Vigilance, Market Surveillance and Control, Licensing establishment, Regulatory Inspection, Laboratory Testing and Clinical Trials Oversight. The legal framework provides adequate powers to the Rwanda FDA to ensure the quality, safety and efficacy of medicines on the market and the scope of products to be regulated is well defined.

However, some challenges hinder the implementation of key regulatory functions to ensure the quality of medicines that should be resolved for the benefit of all stakeholders and the public in general.

Therefore, the capacity of Rwanda FDA should be strengthened to ensure the availability of medicines of assured quality, safety and efficacy throughout the healthcare supply chain. This suggests a need for availing sufficient staff to perform key regulatory functions; avail screening technologies for SF medicines at the main ports of entry; equip the quality control laboratory with adequate equipment; automate all key regulatory functions to ensure transparency, accountability and effective communication with all stakeholders; implement a comprehensive quality management system and plan regulatory inspections based on quality risk management (QRM) approach for effective use of available resources.

## Data Availability

All materials and data are available from the corresponding author without any restriction.

## Abbreviations

ADRs: Adverse drug reactions
AEFI: Adverse events following immunization
LMICs: Low- and middle-income countries
MTaPS: The USAID Medicines, Technologies, and Pharmaceutical Services
NI: Not implemented
OI: Ongoing implementation
PI: Partial implemented
QMS: Quality Management System
QRM: Quality risk management
Rwanda FDA: Rwanda Food and Drugs Authority
SF: Substandard and falsified
WHO: World Health Organization

## References

[1] Orubu ESF, Ching C, Zaman MH, Wirtz VJ (2020). Tackling the blind spot of poor-quality medicines in Universal Health Coverage. J Pharm Policy Pract.

[2] World Health Organization. Ten years in public health, 2007–2017: report by Dr Margaret Chan, Director-General, World Health Organization; 2017. https://apps.who.int/iris/handle/10665/255355. Accessed 09 Aug 2022.

[3] World Health Organization (WHO). A study on the public health and socioeconomic impact of substandard and falsified medical products. Geneva: World Health Organization; 2017. https://www.who.int/publications/i/item/9789241513432. Accessed 26 June 2022.

[4] Ozawa S, Evans DR, Bessias S, Haynie DG, Yemeke TT, Laing SK (2018). Prevalence and estimated economic burden of substandard and falsified medicines in low- and middle-income countries. JAMA Netw Open.

[5] World Health Organization. WHO Global surveillance and monitoring system for substandard and falsified medical products. Geneva: World Health Organization; 2017. http://www.who.int/medicines/regulation/ssffc/publications/GSMS_Report.pdf?ua=1. Accessed 26 June 2022.

[6] Johnston A, Holt DW (2014). Substandard drugs: a potential crisis for public health. Br J Clin Pharmacol.

[7] Ozawa S, Higgins CR, Yemeke TT, Nwokike JI, Evans L, Hajjou M (2020). Importance of medicine quality in achieving universal health coverage. PLoS ONE.

[8] World Health Organization (WHO). Who global benchmarking tool (GBT) for evaluation of national regulatory systems. 2020. https://www.who.int/publications/i/item/9789240020245. Accessed 28 June 2022.

[9] Ndomondo-Sigonda M, Miot J, Naidoo S, Dodoo A, Kaale E (2017). Medicines regulation in Africa: current state and opportunities. Pharm Med.

[10] Arik M, Bamenyekanye E, Fimbo A, Kabatende J, Kijo AS, Simai B (2020). Optimizing the East African community’s medicines regulatory harmonization initiative in 2020–2022: a roadmap for the future. PLoS Med.

[11] Ncube BM, Dube A, Ward K (2021). Establishment of the African Medicines Agency: progress, challenges and regulatory readiness. J Pharm Policy Pract BioMed Cent.

[12] Government of Rwanda. Law N^o^ 003/2018 of 09/02/2018 Establishing Rwanda Food and Drugs Authority and determining its mission, Organisation and Functioning. Gazette. 2018;56–92. https://www.rwandafda.gov.rw/index.php?eID=dumpFile&t=f&f=35810&token=e29060b74d1b91536aa1c657a6ce0bda922a282d.

[13] Kaddu G, D’Amore E, Clark A, Nkansah P. Strengthening regulatory systems to improve medical product quality in low-and middle-income countries. 2018.

[14] World Health Organization (WHO). Manual for benchmarking of the national regulatory system of medical products and formulation of institutional development plans. 2021;1–81. https://www.who.int/publications/m/item/Benchmarking_manual_V2_09Mar2021. Accessed 04 July 2022.

[15] Government of Rwanda. Prime Minister’s Order N° 162/03 of 21/12/2020 determining organizational structure of Rwanda Food and Drugs Authority. 2020. https://gazettes.africa/archive/rw/2020/rw-government-gazette-dated-2020-12-21-no-41.pdf. Accessed 29 June 29 2022.

[16] Kulkarni B, Vemuri R (2015). Role of Quality Management System (QMS) for effective regulatory compliance. Appl Clin Res Clin Trials Regul Aff.

[17] WHO. WHO guideline on the implementation of quality management systems for national regulatory authorities. 2020. https://www.who.int/docs/default-source/medicines/norms-and-standards/guidelines/prequalification/trs1025-annex13.pdf?sfvrsn=2fe3b8d_2

[18] World Health Organization (WHO). Assessment of medicines regulatory systems in sub-Saharan African countries. An overview of findings from 26 assessment reports. 2010;210.

[19] World Health Organization (WHO). Implementing quality management systems in national regulatory authorities. 2021. https://www.who.int/publications/i/item/9789240022379. Accessed 04 July 2022.

[20] Mashingia JH, Ahonkhai V, Aineplan N, Ambali A, Angole A, Arik M (2020). Eight years of the East African Community Medicines Regulatory Harmonization initiative: implementation, progress, and lessons learned. PLoS Med.

[21] USAID/MTaPS. Improving access to maternal, newborn, and child health products in low- and middle-income countries: considerations for effective registration systems. 2021. https://www.mtapsprogram.org/wp-content/uploads/2021/03/MTaPS-Technical-brief_-Registration-of-MNCH.

[22] Ndomondo-Sigonda M, Miot J, Naidoo S, Masota NE, Ng’andu B, Ngum N (2021). Harmonization of medical products regulation: a key factor for improving regulatory capacity in the East African Community. BMC Public Health.

[23] Chattu VK, Dave VB, Reddy KS, Singh B, Sahiledengle B, Heyi DZ (2021). Advancing african medicines agency through global health diplomacy for an equitable pan-African universal health coverage: a scoping review. Int J Environ Res Public Health.

[24] Barry A, Olsson S, Minzi O, Bienvenu E, Makonnen E, Kamuhabwa A (2020). Comparative assessment of the national pharmacovigilance systems in East Africa: Ethiopia, Kenya, Rwanda and Tanzania. Drug Saf.

[25] Isah AO, Pal SN, Olsson S, Dodoo A, Bencheikh RS (2012). Specific features of medicines safety and pharmacovigilance in Africa. Ther Adv Drug Saf.

[26] Ampadu HH, Hoekman J, de Bruin ML, Pal SN, Olsson S, Sartori D (2016). Adverse drug reaction reporting in Africa and a comparison of individual case safety report characteristics between Africa and the rest of the world: analyses of spontaneous reports in VigiBase®. Drug Saf.

[27] Pharmacovigilance infrastructure and post-marketing surveillance system capacity building for regional medicine regulatory harmonization in East Africa (PROFORMA). https://proforma.ki.se/. Accessed 08 Aug 2022.

[28] Vickers S, Bernier M, Zambrzycki S, Fernandez FM, Newton PN, Caillet C (2018). Field detection devices for screening the quality of medicines: a systematic review. BMJ Glob Health.

[29] Mellisa T, Wonder G, Gaparayi Patrick TD. Assessment of the Medicines Regulatory System in Angola: Report. 2013. http://siapsprogram.org/publication/assessment-of-the-medicines-regulatory-system-in-angola-report/.

[30] World Health Organization (WHO). Resolution on the regional strategy on regulation of medical products in the African Region 2016–2025 (Documents AFR/RC66/13 and AFR/RC66/R2). 2016. https://apps.who.int/iris/bitstream/handle/10665/251491/AFR_RC66_R2-eng.pdf?sequence=1&isAllowed=y.

[31] World Health Organization. WHO good practices for pharmaceutical quality control laboratories. WHO Tech. Rep. Ser. 2010. https://www.who.int/publications/m/item/who-good-practices-for-pharmaceutical-quality-control-laboratories---trs-957---annex-1. Accessed 29 June 2022.

